# Pulmonary thromboembolism: new diagnostic imaging techniques

**DOI:** 10.1590/0100-3984.2017.0191

**Published:** 2018

**Authors:** Julia Noschang, Marcos Duarte Guimarães, Diogo Fábio Dias Teixeira, Juliana Cristina Duarte Braga, Bruno Hochhegger, Pablo Rydz Pinheiro Santana, Edson Marchiori

**Affiliations:** 1 MD, Resident in Radiology in the Department of Imaging of the A.C.Camargo Cancer Center, São Paulo, SP, Brazil.; 2 MD, PhD, Radiologist in the Department of Imaging of the A.C.Camargo Cancer Center, São Paulo, SP, Brazil.; 3 Medical Student at the Universidade Anhembi Morumbi (UAM), São Paulo, SP, Brazil.; 4 PhD, Adjunct Professor of Radiology at the Universidade Federal de Ciências da Saúde de Porto Alegre (UFCSPA), Porto Alegre, RS, Brazil.; 5 MD, Radiologist at Medimagem - Hospital Beneficência Portuguesa and for Grupo Fleury, São Paulo, SP, Brazil.; 6 Full Professor of Radiology at the Universidade Federal do Rio de Janeiro (UFRJ), Rio de Janeiro, RJ, Brazil.

**Keywords:** Pulmonary embolism, Tomography, emission-computed/methods, Tomography, X-ray computed/methods, Computed tomography, dual-energy scanned projection, Ventilation-perfusion ratio

## Abstract

The accurate diagnosis of pulmonary thromboembolism is essential to reducing the morbidity and mortality associated with the disease. The diagnosis of pulmonary thromboembolism is challenging because of the nonspecific nature of the clinical profile and the risk factors. Imaging methods provide the definitive diagnosis. Currently, the imaging method most commonly used in the evaluation of pulmonary thromboembolism is computed tomography. The recent development of dual-energy computed tomography has provided a promising tool for the evaluation of pulmonary perfusion through iodine mapping. In this article, we will review the importance of diagnosing pulmonary thromboembolism, as well as the imaging methods employed, primarily dual-energy computed tomography.

## INTRODUCTION

Pulmonary thromboembolism (PTE) is a common clinical entity that results in morbidity and mortality in a large number of patients. PTE is the third leading cause of cardiovascular death in the United States^(^^1^^)^, with an incidence rate of 0.5-1.0 per 1000 person-years^(^^2^^)^. Studies on the epidemiology of PTE in Brazil are rare. Most such studies analyze autopsy data and show that, under those conditions, the prevalence of PTE ranges from 3.9% to 16.6%^(^^3^^,^^4^^)^. Therefore, although PTE occurs frequently, the diagnosis continues to be a great clinical challenge, because the same signs and symptoms are present in a large number of diseases, as well as because the risk factors associated with PTE are nonspecific.

The diagnosis of PTE is based on the following^(^^1^^)^: the clinical pre-test probability; the D-dimer level; and the findings in the image. The imaging methods currently available for the diagnosis are conventional chest X-ray, pulmonary angiography, ventilation-perfusion lung scintigraphy, magnetic resonance imaging (MRI) of the chest, computed tomography (CT) of the chest^(^^5^^)^, and dual-energy CT (DECT), which is the method developed most recently^(^^6^^)^.

Although the concept of DECT originated in 1970, it was not incorporated into clinical practice until recently, when advancements in CT scanner technology, including single- and dual-source configurations, made it feasible^(^^7^^)^. In imaging of the chest, DECT has been used for the clinical evaluation of pulmonary emphysema, pulmonary nodules, ground glass opacities, lung cancer, as well as for the diagnosis of acute PTE, chronic PTE, and chronic thromboembolic pulmonary hypertension^(^^8^^)^. In this review article, we analyze the importance of the diagnosis of PTE, as well as the available imaging methods, with an emphasis on DECT.

## EVALUATION OF PATIENTS WITH PTE

The diagnostic evaluation of a patient with suspected PTE begins with the analysis of the pre-test probability and association with the D-dimer level. Such evaluations are typically based on one of the two most extensively validated criteria-the Wells score^(^^9^^)^ and the Geneva score^(^^10^^)^-which are detailed in Tables 1 and 2, respectively. The presence of risk factors is an essential condition for clinical suspicion. The following are the main risk factors for PTE^(^^11^^)^: surgical or nonsurgical trauma; age greater than 40 years; venous thromboembolism; immobilization; malignant disease; cardiac insufficiency; myocardial infarction; paralysis of the lower limbs; obesity; varicose veins; high estrogen level; childbirth; and chronic obstructive pulmonary disease.

**Table 1:** Main risk factors for PTE - Wells Score.

Criteria		Score
Suspected venous thromboembolism		3
Alternative diagnosis less likely that PTE		3
Heart rate > 100 bpm		1.5
Immobilization or surgery in the last four weeks		1.5
History of venous thromboembolism or PTE		1.5
Hemoptysis		1
Malignancy		1
Score	Probability of PTE	Risk
0-2	3.6%	Low
3-6	20.5%	Moderate
> 6	66.7%	High

**Table 2:** Main risk factors for PTE - Geneva Score.

Criteria	Score
Risk factors	
> 65 years of age	1
History of pulmonary venous thromboembolism or PTE	3
History of surgery or fracture at least one month prior	2
Active malignancy	2
Symptoms	
Unilateral arm pain	3
Hemoptysis	2
Signs	
Heart rate of 75-94 bpm	3
Heart rate > 94 bpm	5
Pain on palpation of the veins of the arm or edema	4
Score	Risk
0-3	Low
4-10	Intermediate
> 10	High

Another notable situation is the identification of PTE as an incidental finding. In the general population, PTE is detected incidentally in 1.0-1.5% of imaging studies performed for another purpose^(^^12^^)^. It is believed that PTE can be overlooked in such imaging studies because of the small caliber of the arteries involved, as well as because the underlying disease can have thoracic manifestations, which can confuse the radiologist in the complete evaluation of the pulmonary arteries^(^^13^^)^.

## IMAGING EVALUATION OF PATIENTS WITH SUSPECTED PTE

### Conventional chest X-ray

In patients with PTE, conventional chest X-ray can show nonspecific signs. In 1940, Hampton et al.^(^^14^^)^ described the classic radiographic appearance of pulmonary infarction, which became known as Hampton's hump, a wedge-shaped peripheral consolidation with its base facing the pleural surface. Subsequently, a number of imaging patterns were associated with pulmonary infarction: peripheral consolidation without air bronchogram; aseptic excavation; and consolidation containing radiolucent areas (distinct from air bronchograms). Other signs have also been reported to be associated with PTE^(^^1^^)^, primarily the Westermark sign (hypovolemia in the region of the lung irrigated by the occluded vessel); elevation of the ipsilateral hemidiaphragm; proximal enlargement of the pulmonary artery; atelectasis; and pleural effusion.

The most common abnormalities are atelectasis, small pleural effusions, and localized reduction in the peripheral blood flow, with or without distension of the proximal vessels. Atelectasis results in a loss of volume in the lower zones, caused by ischemia, which induces surfactant deficiency^(^^15^^)^. Although conventional chest X-ray findings are abnormal in most cases of PTE, they are normal in 40% of cases^(^^16^^)^.

### Pulmonary angiography

**Figure 1:**
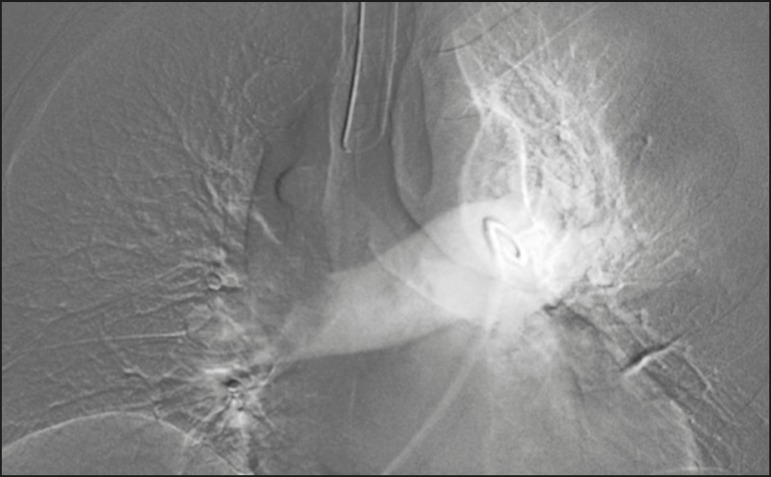
Pulmonary arteriography identifying bilateral pulmonary trunk filling defect, with preservation of the contrast opacification only in the apical posterior segment.

Pulmonary angiography is an invasive diagnostic method in which an intravenous catheter is introduced into the proximal pulmonary artery and the contrast medium is injected rapidly. The technique offers high spatial resolution, allowing direct evaluation of the pulmonary arterial tree. Filling defects within the contrast column are typical findings in PTE (Figure 1). Although pulmonary angiography is considered the gold standard method, it can lead to complications, mainly anaphylaxis, contrast-induced nephrotoxicity, cardiac events, and pulmonary complications^(^^11^^)^. Among patients undergoing pulmonary angiography, fatal complications occur in 0.5%, major complications (life-threatening events that do not respond to therapy and require intensive care or prolonged hospitalization) occur in 1%, and minor complications (requiring long-term follow-up but regressing spontaneously without residual damage) occur in 5%^(^^17^^)^.

### Ventilation-perfusion lung scintigraphy

**Figure 2:**
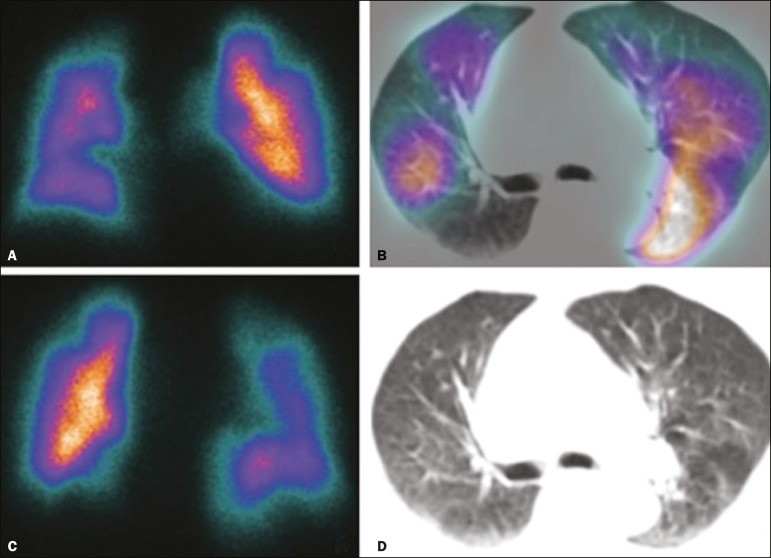
Pulmonary perfusion scintigraphy in coronal sections (**A,B**) and SPECT/CT in axial sections (**C,D**) in a 77-year-old male patient, demonstrating multiple wedge-shaped perfusion defects bilaterally, without parenchymal alterations, indicating a high probability of PTE.

Ventilation-perfusion lung scintigraphy is aimed at identifying ventilation in regions without perfusion, at a location distal to obstructing emboli (Figure 2), which is suggestive of a diagnosis of PTE. On the basis of the scintigraphic findings, the probability of embolism is classified as follows: high; intermediate; low; very low; or nonexistent. Scintigraphy findings indicating a high probability confirm the diagnosis of PTE, whereas those indicating a very low or nonexistent probability allow the diagnosis to be excluded. However, the limitation of ventilation-perfusion scintigraphy is related to a high number of patients in whom the findings are not conclusive. In the Prospective Investigation of Pulmonary Embolism Diagnosis (PIOPED) study, ventilation-perfusion scintigraphy was unable to establish or exclude the diagnosis of PTE in two thirds of the patients who underwent the examination^(^^18^^)^.

The anatomical data obtained with single-photon emission CT (SPECT)/CT can be associated with the functional data obtained with scintigraphy. There have been few studies on the utility of SPECT/CT in the diagnosis of PTE, and there are no definitive recommendations regarding the method. However, it is believed that SPECT/CT has a high (99%) accuracy for the diagnosis, with a sensitivity of 97-100% and a specificity of 83-100%^(^^19^^,^^20^^)^.

### MRI of the chest

The development of new MRI techniques, which reduce acquisition time, increase resolution and decrease movement artifacts, has allowed the use of this method for the diagnosis of PTE. The sensitivity and specificity of MRI for the evaluation of PTE are 78-100% and 95-100%, respectively^(^^21^^,^^22^^)^. 

The PIOPED III study evaluated the accuracy of gadolinium-enhanced MRI. The authors found that, when the technique was appropriate, MRI showed a sensitivity and specificity of 78% and 99%, respectively, for the diagnosis of PTE. However, the mean proportion of tests considered technically suboptimal was 25%. Therefore, when the technically suboptimal images were included in the analysis, there was a 57% reduction in sensitivity^(^^23^^)^.

**Figure 3:**
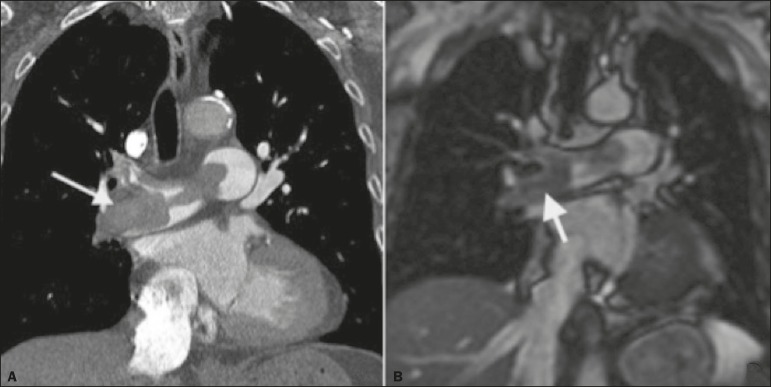
Correlation between a coronal CT slice (**A**) and a T2-weighted fast-spin-echo coronal MRI sequence (**B**) showing a filling defect at the pulmonary artery bifurcation.

In another study, the concordance between multiple-detector computed tomography and MRI was found to be greater when multiple MRI techniques (real-time MRI, MRI angiography, and MRI perfusion scintigraphy) were analyzed jointly; the sensitivity and specificity of the combined MRI protocol for the diagnosis of pulmonary embolism were found to be 100% and 93%, respectively^(^^24^^)^. Therefore, MRI, because of its high sensitivity and specificity, is an effective non-ionizing alternative for the diagnosis of PTE (Figure 3).

### Chest CT

Chest CT has been the object of a series of studies in the radiology literature of Brazil^(^^25^^-^^31^^)^. It is increasingly used for the diagnosis of PTE, being considered the standard method for this diagnosis at many facilities^(^^32^^)^. However, the method is of limited utility for the diagnosis of small peripheral emboli, reportedly missing 53% of cases of peripheral or subsegmental PTE^(^^33^^)^.

For the diagnosis of PTE, chest CT is performed with the use of intravenous iodinated contrast medium. For intravenous access, an 18- or 20-gauge catheter is inserted, preferentially into an antecubital vein. The contrast medium is injected in a volume of 135 mL, at an infusion rate of 4 mL/s. The images are displayed in three different gray scales: a lung window with a width/level of 1500/600 Hounsfield Unity (HU); a mediastinal window with a width/level of 400/40 HU; and specific window for pulmonary embolism with a width/level of 700/100 HU^(^^34^^)^.

**Figure 4:**
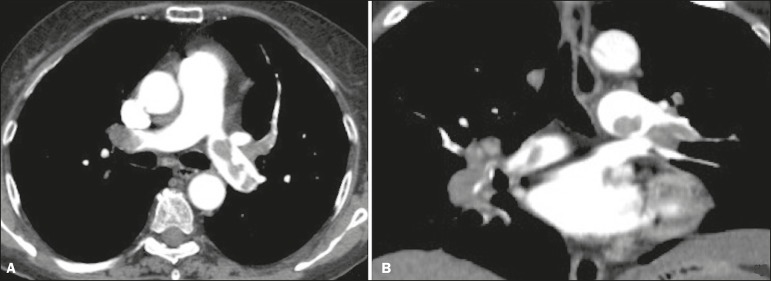
Acute PTE in a 62-year-old female patient. CT slices, in axial and coronal views (**A** and **B**, respectively), showing an extensive irregular filling defect in the right and left pulmonary arteries, extending to its segmental branches.

**Figure 5:**
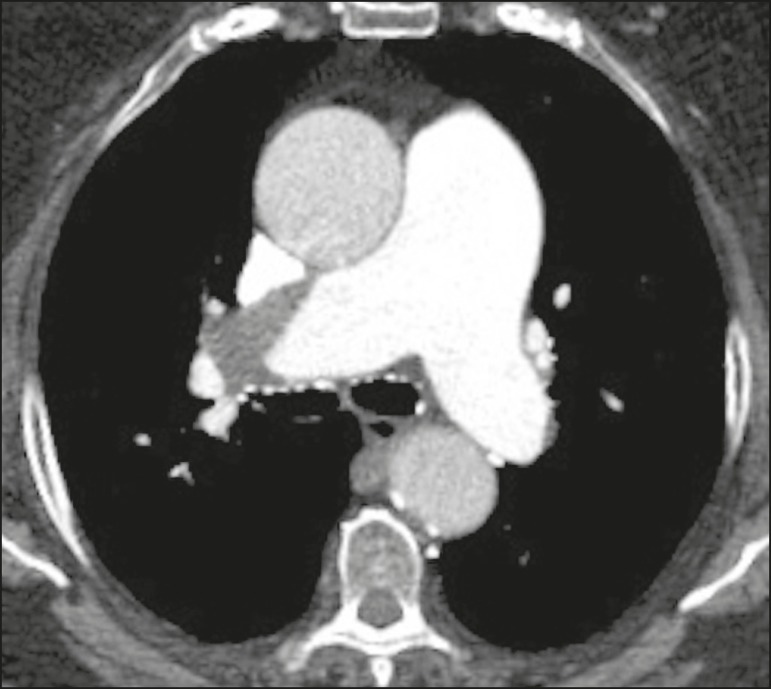
Chronic PTE in an 86-year-old female patient with a history of breast cancer. Axial CT scan showing a filling defect with obtuse margins in the right pulmonary artery, with patent flow in the distal bed.

The diagnosis of PTE by CT is based on the identification of intraluminal filling defects on contrast-enhanced images. The diagnostic criteria for acute PTE include the following (Figure 4): arterial occlusion with filling defects throughout the lumen, the artery being occluded and its diameter being increased in comparison with the adjacent vessels; partial contrast filling defect, producing the "polo mint" sign in images perpendicular to the long axis of the vessel and the "tram-track" sign in images acquired along that axis; and peripheral filling defects forming acute angles with the arterial wall. The following are the diagnostic criteria for chronic PTE (Figure 5): complete occlusion of a vessel that is of a smaller caliber than the adjacent vessel; a crescent-shaped peripheral filling defect forming an obtuse angle with arterial wall; uneven flow of the contrast agent, often associated with arterial recanalization; and the presence of a "web" within a contrast-filled artery^(^^34^^)^.

## OVERVIEW OF DECT

### Basic principles

The DECT method is based on the premise that materials behave differently when exposed to X-ray photons of different energies. Therefore, it allows materials with different molecular compositions to be distinguishing on the basis of their attenuation^(^^35^^,^^36^^)^.

The development of DECT was made possible by modifications to conventional CT scanners, the various modifications resulting in different DECT systems. The first involved the use of single source CT scanners with the acquisition of two sequential sets of images with different photon spectra (for example, 80 kVp followed by 140 kVp). One significant limitation of sequential dual-energy CT is alteration of the post-reconstruction contrast enhancement over time, which compromises the measurement of the decomposition of the material in question. The simultaneous acquisition of images at two different energies eliminates this limitation, and post-processing algorithms allow the materials to be differentiated. Currently, there are two DECT techniques that have been approved by the US Food and Drug Administration for clinical use^(^^35^^,^^37^^)^: dual-source TC scanners; and single-source CT scanners with rapid voltage switching.

The increased clinical use of DECT was accompanied by concern about the amount of radiation involved in the use of the technique. A number of studies have compared the use of conventional CT with that of DECT and have concluded that neither method involves greater or lesser exposure to radiation than the other. Few of those studies focused on the normalization of image quality, the signal-to-noise ratio, or the dose-length product^(^^38^^)^. However, it is believed that the DECT is feasible without additional radiation, and there is no significant difference in the image noise, because the contrast-to-noise ratio can be improved by optimizing DECT reconstructions^(^^32^^)^. It is also of note that the studies evaluating single-source CT with rapid voltage switching have produced inconclusive results^(^^38^^)^.

### Post-processing techniques

Two approaches are used in order to obtain post-processing information. One is to subtract equivalent projections and apply filtering to earlier projections to reconstruct the differences in the spectral information. Another way is to reconstruct standard CT images into voxels (in HU) and use post-processing algorithms to extract specific spectral information based on the differences between the corresponding voxels. Currently, the latter is the most commonly used approach, with an image reconstruction system characterized by low and high kilovoltage, as well as a series of reproductions with the weighted mean^(^^37^^)^.

The main post-processing image reconstruction techniques used in DECT are iodine maps, virtual non-contrast-enhanced images, and virtual monochromatic images^(^^35^^,^^36^^)^. For the diagnosis of PTE, iodine mapping allows the visualization of the distribution and amount of the substance in the pulmonary parenchyma, which is related to pulmonary perfusion. Dual-source DECT uses a decomposition algorithm based on three materials with known low- and high-energy X-ray absorption properties (iodine, soft tissues, and fat). The estimated quantity of each material is calculated based on its attenuation profile at different energy levels; thus, an iodine-specific map is generated by determining the amount of iodine in the tissue (in HU). Single-source DECT uses a decomposition algorithm based on two materials in the projection space. The low- and high-energy attenuation values for the two materials selected (water and iodine) are mathematically transformed to calculate the values that would be required to determine those levels of attenuation^(^^35^^)^.

For the evaluation of iodine, the images can be displayed quantitatively, as a grayscale image, or qualitatively, in the form of a colored map. Both forms provide an indirect assessment of the microvascular environment of the underlying tissue. In dual-source DECT, the quantity of iodine is expressed in HU, the mean value representing the degree of enhancement. In the single-source method, the quantity of iodine is expressed in milligrams per milliliter. Another way of evaluating pulmonary perfusion through CT is the technique of subtracting a post-contrast phase from a pre-contrast phase, creating iodine maps similar to those provided by DECT^(^^39^^)^.

### Diagnosis of PTE

**Figure 6:**
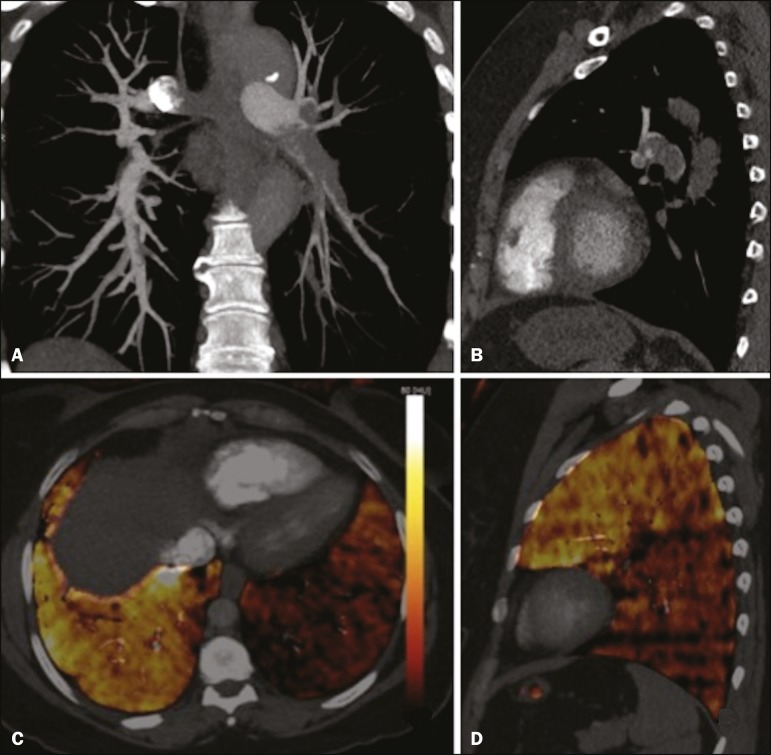
CT slices, in coronal and sagittal views (**A** and **B**, respectively), showing extensive filling defects affecting the pulmonary artery branches, mainly in the left lower lobe. Dual-energy CT, in axial and sagittal views (**C** and **D**, respectively), demonstrating na extensive perfusion defect in the left lower lobe due to acute PTE.

**Figure 7:**
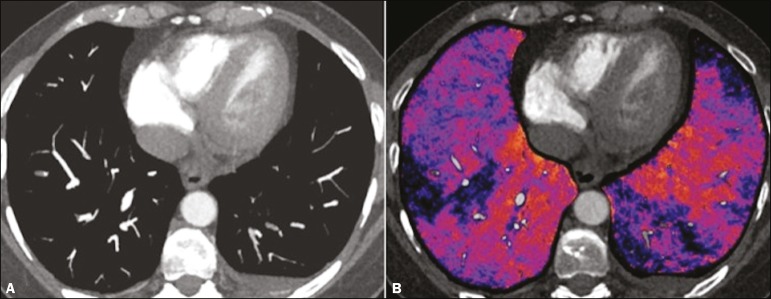
Axial CT slice (**A**) and iodine map created by the subtraction technique (**B**) showing bilateral filling defects in the subsegmental branches in correlation with the associated perfusion defects.

To the morphological information provided by CT, the use of DECT adds data regarding pulmonary parenchymal perfusion through iodine mapping, which allows the visualization of the iodine distribution in the parenchyma^(^^36^^)^, as depicted in Figure 6. The use of iodine mapping in DECT is expected to improve the accuracy of the diagnosis of PTE, especially for segmental and subsegmental PTE (Figure 7), because small segmental and subsegmental thrombi may not be detected by CT in clinical radiology practice.

Studies evaluating the use of DECT have shown that it is superior to conventional CT for the detection of PTE. One experimental study showed that the sensitivity for the detection of PTE was 89% for DECT, compared with 67% for conventional CT^(^^40^^)^. In agreement with those findings, clinical studies have also demonstrated benefits of DECT use. In one such study, the per-patient sensitivity and specificity for the detection of PTE reached 100%^(^^41^^)^, with a sensitivity of 84.6-100% and a specificity of 93.3-100%. However, for the detection of segmental and subsegmental PTE, DECT presents a sensitivity of 60.0-82.9% and a specificity of 99.5-99.8%^(^^41^^,^^42^^)^.

It is noteworthy that DECT shows high rates of interobserver and intraobserver agreement rates, demonstrating good applicability of the method in clinical practice^(^^43^^)^. However, the contact of the pulmonary segments with the upper mediastinum or heart chambers is considered a limiting factor for the appropriate evaluation of PTE by DECT^(^^44^^)^.

The perfusion defects demonstrated by DECT have good agreement with the findings of ventilation-perfusion scintigraphy. In a study comparing DECT with scintigraphy in patients who underwent both techniques, with a mean interval of three days between the two examinations, the per-patient accuracy of the diagnosis showed a sensitivity of 75%, a specificity of 80%, and a negative predictive value of 93%. When assessed by lung segment, the sensitivity was 83%, the specificity was 99%, and the negative predictive value was 93%^(^^45^^)^. In another study, comparing pulmonary perfusion with DECT and SPECT/CT, DECT demonstrated 100% sensitivity and specificity for acute PTE, whereas the combination of SPECT/CT and ventilation scintigraphy had a sensitivity of 85.7% and a specificity of 87.5%^(^^46^^)^.

Despite the fact that there are a limited number of studies comparing pulmonary perfusion demonstrated by iodine mapping with that demonstrated by MRI, the results are not satisfactory. To date, there appears to be no significant correlation between the two methods, which have shown only a moderate level of visual correlation^(^^47^^)^.

Regarding DECT, the possibility of a correlation between the extent of the perfusion defect and the prognosis should be highlighted, given that a significant increase in the volume of the perfusion defect (35% ± 11% versus 23% ± 10%, *p* = 0.002) has been associated with adverse clinical outcomes^(^^48^^)^.

### Limitations

Although DECT is a promising tool for the diagnosis of PTE, there are some challenges for its use in clinical practice. The following factors have been shown to limit the use of DECT:

- Scanner-related: limited access to DECT; high cost; relatively long image processing time; and smaller field of view of the B tube, which may not include the peripheral portion of the thorax, thus preventing DECT from being used for dual-energy post-processing.

- Patient-related: obesity can which can increase image noise, compromising the structural and functional analysis, and, in some cases, patient weight exceeds the allowable limit of the DECT scanner. 

- Interpretation: there are a limited number of radiologists who are familiar with the technique; and the terminology has yet to be standardized.

### Artifacts in image interpretation

Artifacts in the iodine concentration map images should be considered, in order to avoid misdiagnosis. Such artifacts are related to the contrast agent, to its physiological distribution, and to lung disease. Regions with high concentrations of iodinated contrast may cause a beam-hardening effect, resulting in locations with contrast enhancement defects adjacent to an area with high contrast enhancement. The physiological action of gravity should also be recognized, because, in tests performed in the supine position, the anterior regions of the thorax usually show less contrast enhancement. However, nonocclusive thrombi can result in false-negative results, with little enhancement defect in the corresponding region. The reduction in the number of capillaries in elderly patients and in regions with emphysema can also cause enhancement defects^(^^49^^)^.

## CONCLUSION

The DECT technique is capable of identifying perfusion defects with good accuracy, as well as of providing high-resolution images for the evaluation of the morphology of the lung parenchyma. Therefore, it has the potential to increase accuracy and safety in the diagnosis of PTE.
